# Supplementary material for: Temporal regulation of BMP2 growth factor signaling in response to mechanical loading is linked to cytoskeletal and focal adhesion remodeling
Source: Commun Biol. 2024 Aug 30;7:1064. doi: 10.1038/s42003-024-06753-x (PMC11364689; doi:10.1038/s42003-024-06753-x)
Supplement: Supplementary file 2 — Description of Additional Supplementary Materials [file 42003_2024_6753_MOESM2_ESM.pdf]

Description of Additional Supplementary Files

File name: Supplementary Data 1

Description: Modified bioreactor setup used for visualizing local biomaterial straining associated with cyclic compression

File name: Supplementary Data 2

Description: Heat map summarizing changes in gene expression in response to BMP2 stimulation and/or mechanical loading

File name: Supplementary Data 3

Description: Immunhistology of the extracellular matrix composition on the biomaterial scaffold walls

File name: Supplementary Data 4

Description: Co-localization of vinculin, phospho-Paxillin and Integrin alpha V in focal adhesions

File name: Supplementary Data 5

Description: Analysis of BMP2 secretion into the culture medium during 24h bioreactor cultivation

File name: Supplementary Data 6

Description: List of primer sequences used in this work

File name: Supplementary Data 7

Description: Raw western blot scans of blot data shown in Figure 2

File name: Supplementary Data 8

Description: Raw western blot scans of blot data shown in Figure 4

File name: Supplementary Data 9

Description: Raw western blot scans of blot data shown in Figure 5

File name: Supplementary Data 10

Description: Raw western blot scans of blot data shown in Figure 6

File name: Supplementary Data 11

Description: Primary source data of the presented plots
