# Supplementary material for: Temporal regulation of BMP2 growth factor signaling in response to mechanical loading is linked to cytoskeletal and focal adhesion remodeling
Source: Commun Biol. 2024 Aug 30;7:1064. doi: 10.1038/s42003-024-06753-x (PMC11364689; doi:10.1038/s42003-024-06753-x)

Supplementary Data

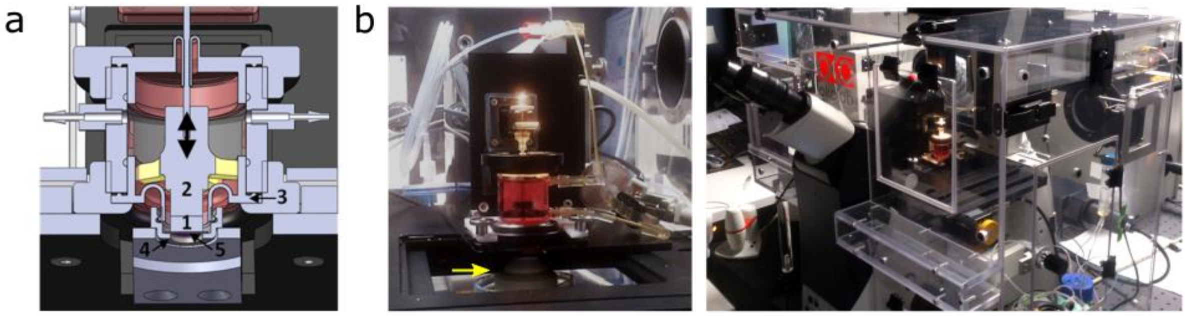

Supplementary Data 1: Modified bioreactor setup for in situ confocal/multi-photon microscopy. (A) Cross-section through the CAD-representation with biomaterial scaffold (1), load-applying plunger (2), Silicone membrane (3), Outer stainless steel ring to seal the silicon membrane against an inner stainless steel ring (not shown) (4) and circular cover glass for optical coupling (water-immersion) to the inverted confocal microscope (5). (B) Overview pictures showing the bioreactor mounted onto the confocal microscope under controlled temperature (microscope housing) and gassing (external gas mixing unit connected to the bioreactor). Yellow arrow indicates the 25x water immersion objective of the inverted microscope.

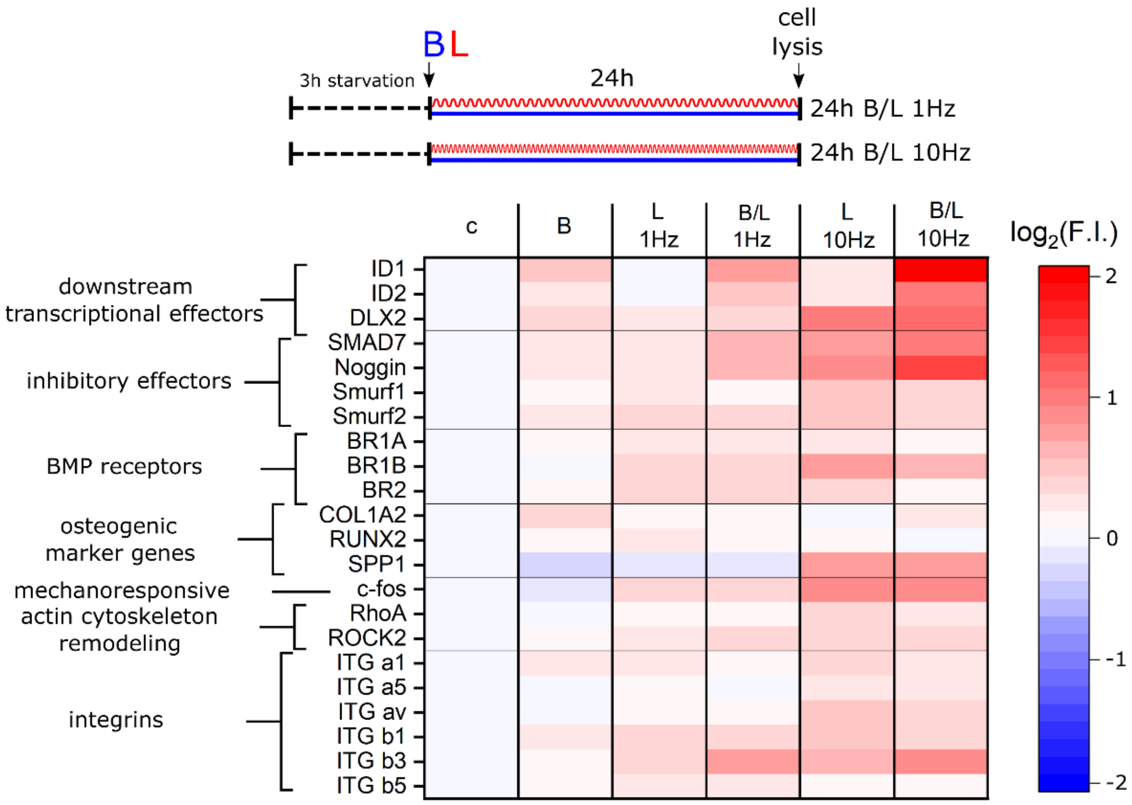

Additional file 2: Heat map summarizing the gene expression changes in response to 24h BMP2 stimulation and/or mechanical loading of 1 Hz or 10 Hz. Induction and reduction of expression is labeled in red and blue respectively, while white indicates no change in comparison to the untreated control (c).

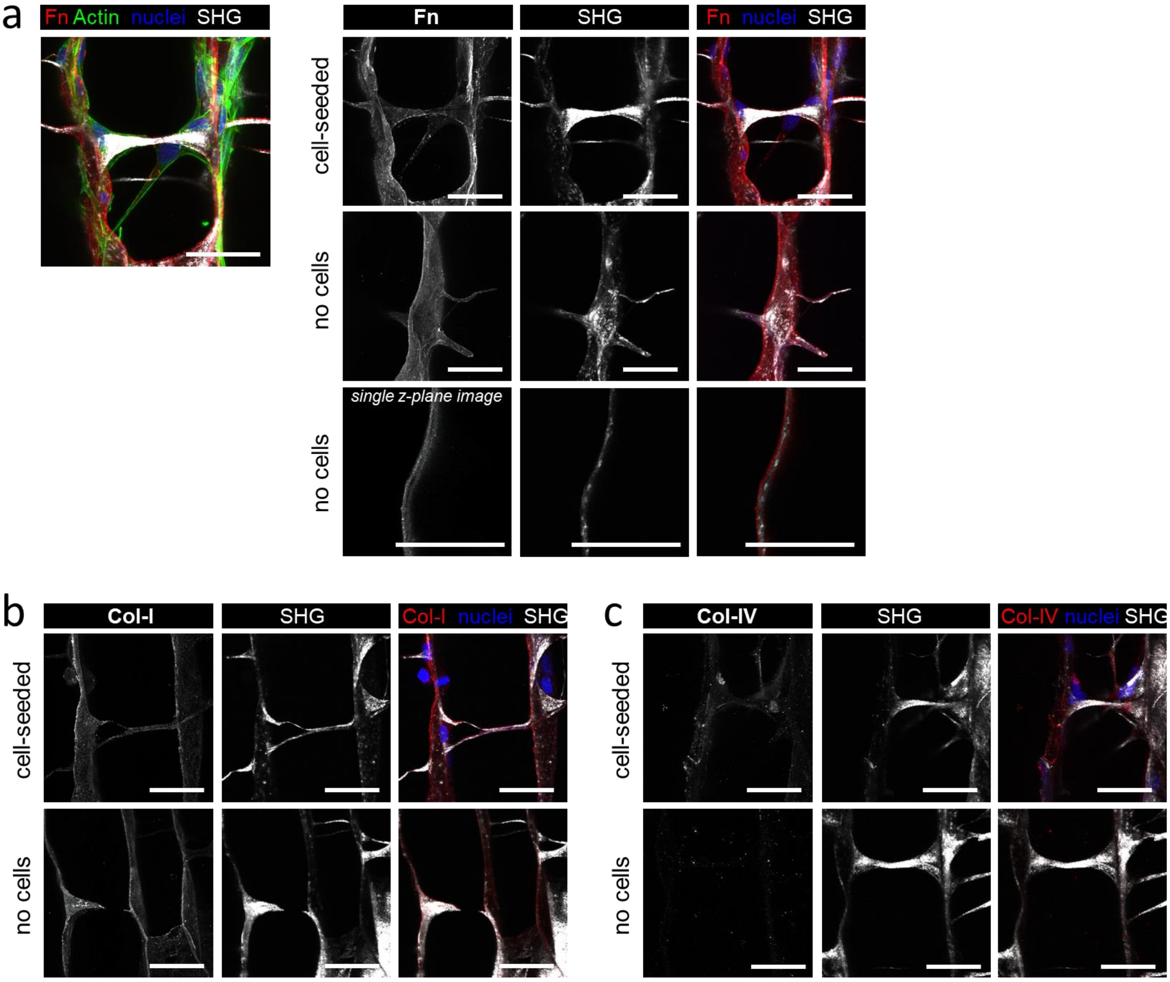

Supplementary Data 3: Immunhistology illustrating the extracellular matrix composition on the biomaterial scaffold walls before and after culture of hFOBs in the material. Condition "no cells" represents scaffolds that were immersed in FBS-containing cell culture medium without cells for 1h at 37°C, "cell-seeded" represents scaffolds in which hFOBs were cultured for 48h at 37°C. Next to the specific ECM molecule of interest (fibronectin "Fn", collagen type I "Col-I" and collagen type IV "Col-IV"), second harmonic imaging was performed to visualize the scaffold wall material consisting of fibrillar collagen-I. (A) Representative image of cells adhering to the scaffold material (top left) and Fn layer detected on the scaffold walls with and without cells in a maximum intensity projection of confocal z-stacks (right). No cell-secreted fibronectin fibers were detected in the scaffold pores. Additional single z-plane images highlight the localization of Fn as thin layers on the two surfaces of the scaffold walls. (B) Corresponding z-stacks of Col-I immunhistology showing that hFOBs did not deposit additional collagen-I fibers on the scaffold walls or in the scaffold pores. (C) A slight increase in Col-IV signal was detected in the "cell seeded" vs. "no cells" condition indicating that cells had deposited a certain amount of col-IV on the scaffold walls over 24h of culture. Scale bar 50µm for all images

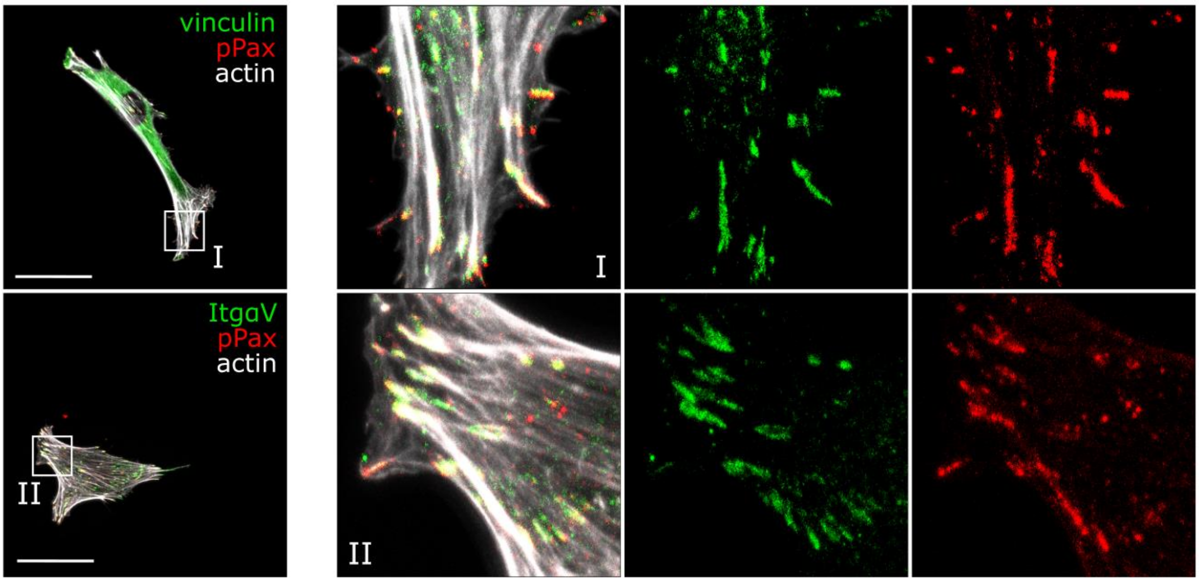

Supplementary Data 4: hFOBs were stained for vinculin (green) and phospho-Paxillin (red) (top panel) or Integrin alpha V (green) and phospho-Paxillin (red) (bottom panel). Zoom-in ROIs illustrate co-localization of signals at cell adhesion points. Scale bar 50µm.

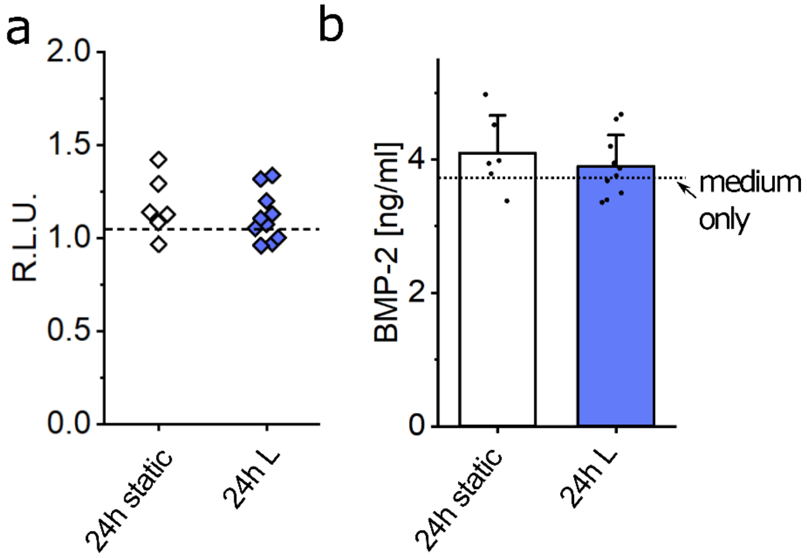

Supplementary Data 5: Analysis of BMP2 secretion into the culture medium during 24h bioreactor cultivation. C2C12 BRE-luc cells were seeded overnight in 24 well plates and serum starved in DMEM containing 0% FCS for 8 hours. After serum starvation, cells were incubated either with collected bioreactor culture medium (24h static, 24h cyclic loading (1Hz, 10%), bioreactor medium only) or with increasing concentrations of BMP2 (standard curve). After 16 hours of treatment, BRE-luciferase activity was assessed by measuring the luciferase activity in cell lysates. (A) Luciferase activity was normalized to protein content. Relative luciferase units (R.L.U.) following background subtraction are shown. (B) BMP2 concentrations were calculated using the BMP2 standard curve. Bar chart shows mean ± SD (n=4).

Supplementary Data 6: Primer sequences

Gene	Protein	Function	Primer sequence 5'→ 3' forward (fwd) and reverse (rev)	
BR1A	BMP type I receptor 1a	BMP signaling	fwd	TTCGATGGCTGGTTTTGCTC
			rev	ACGACGTCTGCTTGAGATGC
BR1B	BMP type I receptor 1b	BMP signaling	fwd	CCTGGAGAATCCCTGAGAGAC
			rev	AGTCCTTTGGACCAGCAGAG
BR2	BMP type I receptor 2	BMP signaling	fwd	GTTGGAGCTGATTGGCCGAG
			rev	TTTACAGCAACTGGACGCTC
c-fos	FBJ murine osteosarcoma viral oncogene homolog	Mechano-sensitive TF	fwd	CAAGCGGAGACAGACCAACT
			rev	AGATCAAGGGAAGCCACAGA
COL1A2	Collagen alpha-2(I) chain	ECM proteins	fwd	AGCCGGAGATAGAGGACCAC
			rev	GGCCAAGTCCAACTCCTTTT
DLX2	Distal-less homeobox 2	BMP target	fwd	GGCGTTTCCAAAAGACTCAA
			rev	CGAAGCACAAGGTGGAGAAG
HPRT1	hypoxanthine phosphoribosyl-transferase 1	House-keeping gene	fwd	TATGGACAGGACTGAACGTC
			rev	TGATGTAATCCAGCAGGTCA
ID1	Inhibitor of DNA binding 1	BMP target	fwd	GCTGCTCTACGACATGAACG
			rev	CCAACTGAAGGTCCCTGATG
ID2	Inhibitor of DNA binding 2	BMP target	fwd	GTGGCTGAATAAGCGGTGTT
			rev	TGTCCTCCTTGTGAAATGGTT
ITGα1	Integrin subunit alpha 1	Cell adhesion	fwd	ACGCTGCTGCGTATCATTCA
			rev	CACCTCTCCCAACTGGACAC
ITGα5	Integrin subunit alpha 5	Cell adhesion	fwd	TGGCCTTCGGTTTACAGTCC
			rev	GGTGCAGTTGAGTCCCGTAA
ITGαv	Integrin subunit alpha v	Cell adhesion	fwd	TCAGCAAGGCAATGCTCCAT
			rev	GAGGGCAAGATCCCGCTTAG
ITGβ1	Integrin subunit beta 1	Cell adhesion	fwd	CTGCGAGTGTGGTGTCTGTA
			rev	CACAGGATCAGGTTGGACCG
ITGβ3	Integrin subunit beta 3	Cell adhesion	fwd	ACCAGTAACCTGCGGATTGG
			rev	TCCGTGACACACTCTGCTTC
ITGβ5	Integrin subunit beta 5	Cell adhesion	fwd	ATACCTGGAACAACGGTGGAG
			rev	AGATCCTCAGGCTGATCCCA
Noggin	Noggin	BMP antagonist	fwd	GCCAGCACTATCTCCACATCC
			rev	GGGTGTTCGATGAGGTCCAC
RUNX2	Runt-related transcription factor 2	Osteogenic marker	fwd	CTCCTACCTGAGCCAGATGA
			rev	CGGGGTGTAAGTAAAGGTGG
Smad7	Smad familiy member 7	BMP signaling	fwd	TGCAACCCCTACCACTTCAGC
			rev	GAGACATGCTGGCGTCTGAG
Smurf1	SMAD specific E3 ubiquitin protein ligase 1	BMP signaling	fwd	AATGAAGATGCGACCGAAAG
			rev	AGCCCGTAATAAGGATTCAGC
Smurf1	SMAD specific E3 ubiquitin protein ligase 2	BMP signaling	fwd	TCCTCGGCTGTCTGCTAACT
			rev	GGGACTGTCAGGCATTCTGT

SPP1	Osteopontin	Osteogenic marker	fwd	TCACCTGTGCCATACCAGTTA
			rev	TCATGGCTTTCGTTGGACTT

Supplementary Data 7: Raw western blot scans for blot data shown in Figure 2

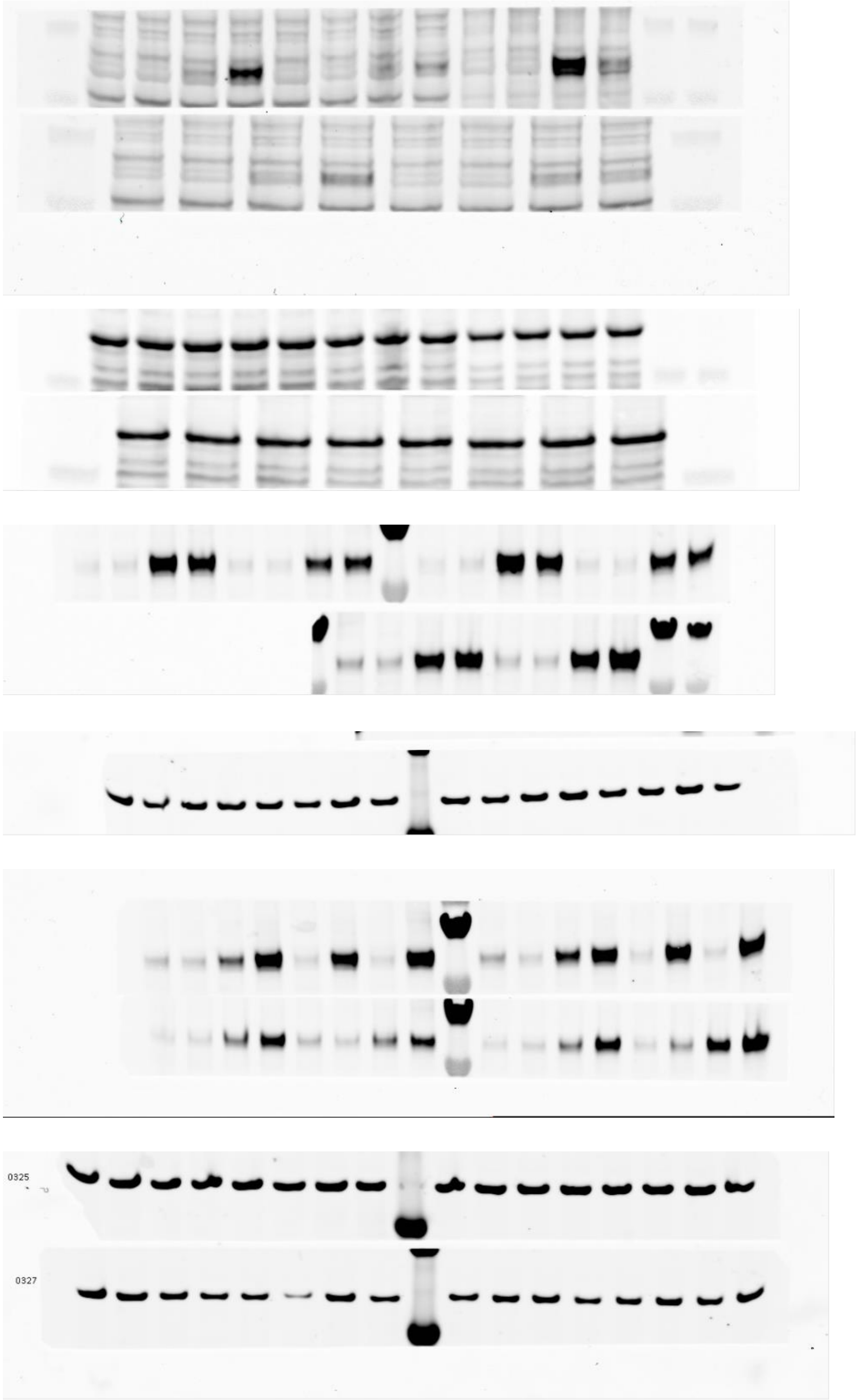

Supplementary Data 8: Raw western blot scans for blot data shown in Figure 4

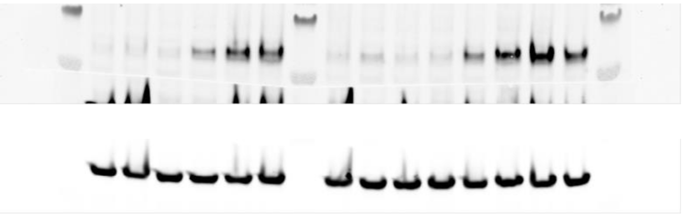

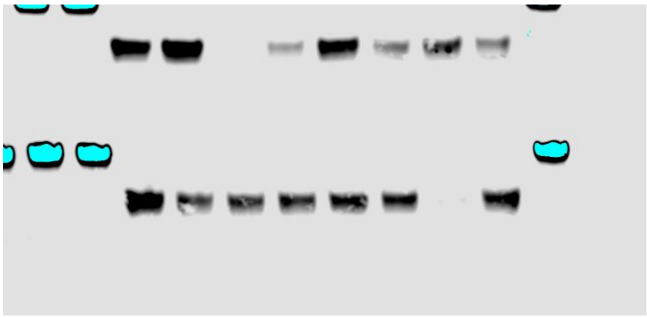

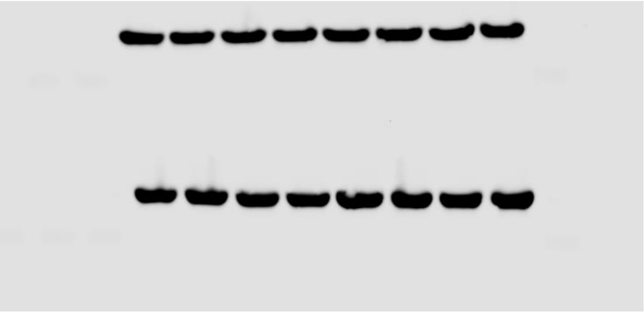

Supplementary Data 9: Raw western blot scans for blot data shown in Figure 5

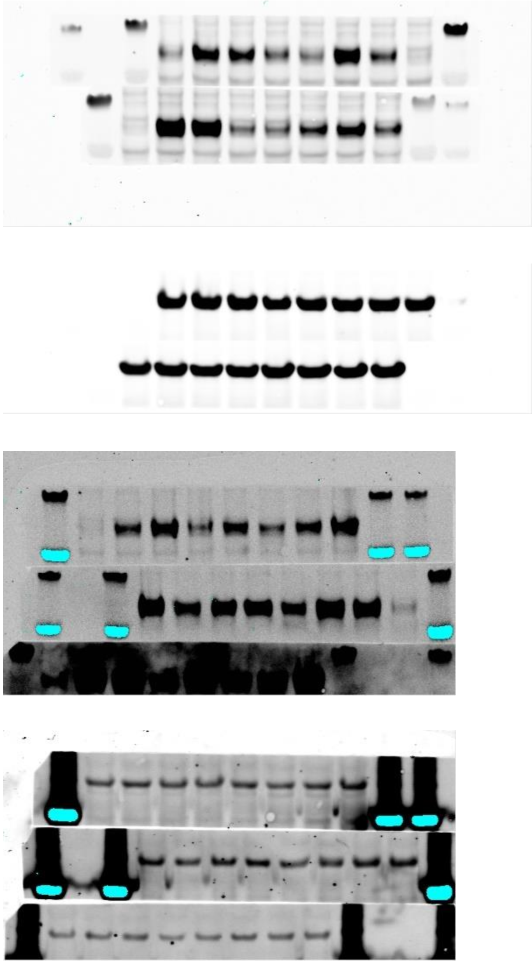

Supplementary Data 10: Raw western blot scans for blot data shown in Figure 6

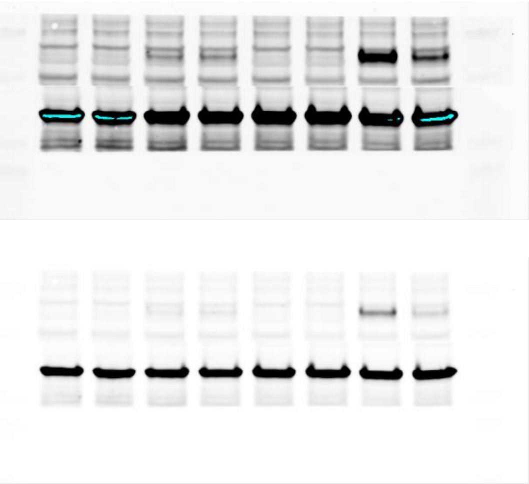

Supplement: Supplementary file 3 — Supplementary Data 1–10 [file 42003_2024_6753_MOESM3_ESM.pdf]
